# A rare case of internal hernia, intussusception and volvulus following gastric bypass: A case report and literature review

**DOI:** 10.1016/j.ijscr.2020.01.060

**Published:** 2020-02-06

**Authors:** Adel Elkbuli, Kristen Santarone, Kyle Kinslow, Mark McKenney, Dessy Boneva

**Affiliations:** a Department of Surgery, Kendall Regional Medical Center, Miami, FL, USA; b University of South Florida, Tampa, FL, USA

**Keywords:** CT, computed tomography, RYGB, roux-en-y gastric bypass, JJ, jejuno-jejunal, ICU, intensive care unit, SBO, small bowel obstruction, Gastric bypass, Intussusception, Internal hernia, Volvulus, Bariatric surgery, Surgical outcomes

## Abstract

**Introduction:**

The Roux-en-y gastric bypass (RYGB) surgery is one of the most common and successful weight loss procedures. Procedure mortality is low, but intestinal complications account for a high percentage of associated morbidity. Internal hernias remain one of the most common complications while volvulus and intussusception are rare.

**Presentation of case:**

A 22-year-old woman with a past surgical history of laparoscopic RYGB six years prior presented with a 12 -h history of abdominal pain. Exploratory laparotomy revealed concomitant volvulus, internal hernia and intussusception at the J-J anastomosis which was reduced without need for bowel resection. Her post-operative course was unremarkable and she was discharged home five days later.

**Discussion:**

Many previous cases of intussusception related to RYGB surgery have required treatment with bowel resection secondary to delayed surgical intervention. Due to high variability in clinical presentation of post-RYGB obstruction, a high index of suspicion is necessary for prompt recognition. Early surgical intervention may prevent the need for bowel resection and improve patient outcomes.

**Conclusion:**

This case represents an unusual complication of RYGB involving intussusception, internal hernia and volvulus that was successfully managed without need for bowel resection due to early identification and surgical intervention.

## Highlights

• Development of a triad of intussusception, volvulus, and internal hernia after RYGB surgery is rare.
• In patients with history of RYGB and acute abdomen, high suspicion for obstructive complications is required to allow for timely diagnosis and early surgical intervention.
• Early surgical intervention of SBO secondary to RYGB was sufficient for preventing bowel necrosis and subsequent bowel resection.

## 1 Introduction

Roux-en-y gastric bypass (RYGB) remains the gold standard for bariatric surgery due to providing safe and sustainable weight loss compared to other operations such as sleeve gastrectomy, gastric banding or biliopancreatic diversion/duodenal switch [[1], [2], [3], [4]]. RYGB involves the creation of a 15−30 mL gastric pouch from the proximal end of the stomach with subsequent anastomosis of the pouch and the Roux limb (approximately 75−150 cm in length). The Roux and biliopancreatic limbs are then anastomosed to create a common channel from the jejunojejunal anastomosis to the cecum (approximately 200 cm or more in length). This can be completed in an antecolic or retrocolic manner via an open or now more commonly laparoscopic approach. Complications include stomal stenosis, anastomotic leak, gastric remnant dilatation, marginal ulcers, gastrogastric fistula, dumping syndrome, biliopancreatic limb obstruction, intussusception, volvulus or internal hernia [5].

Internal hernia is a relatively common cause of bowel obstruction in post-RYGB patients. Development involves a loop of bowel that herniates through a mesenteric defect acquired during the surgery. Such defects are more commonly experienced via a retrocolic approach and therefore higher rates of internal herniation have been shown to occur (3.5–5.6 %). Incidence of internal hernia after RYGB has been shown to decrease when a mesenteric defect is closed intra-operatively and an antecolic approach is utilized (0.9–4.5 %) [6]. Internal herniation occurs most commonly in the Petersen space, a space that exists between the mesentery of the Roux limb and the adjacent transverse mesocolon. This pathology appears to occur regardless of the surgical approach utilized (retrocolic vs. antecolic) [5]. Obstructive presentation is typically non-specific, with mid-epigastric pain, varying physical examination findings and inconclusive imaging studies. CT scan is the most reliable imaging modality and may show “mesenteric swirling” of the mesenteric vessels or fat [7,8]. Due to significant difficulty in identification and diagnosis, exploratory laparoscopy or laparotomy is often the definitive method to ruling out an internal hernia.

Intussusception is a rare complication of RYGB (Female > Male) and has unique development in these patients compared to the standard intussusception in the adult population. In bariatric patients, the intussusception often occurs in a retrograde manner without a “lead point” and is typically located at the common channel to the jejunojejunostomy site. Clinically, patients present with signs and symptoms of small bowel obstruction including abdominal pain, nausea and vomiting with CT Imaging showing a “target sign” approximately 81 % of the time [9]. Treatment for intussusception in these patients involves surgical exploration and reduction.

Volvulus is another cause of bowel obstruction in RYGB patients. Volvulus in this population is known to be caused by placement of a stitch between the roux limb and the excluded stomach in attempt to prevent kinking at the gastrojejunostomy anastomosis. Patient presentation can involve abdominal pain with distention, nausea and vomiting. CT scan of the abdomen and pelvis provides the best imaging, and treatment involves surgical exploration and manual correction.

The 30 day mortality rate for a RYGB is between 0.09-0.12 %, increasing to 4 % if the patient is over the age of 65 [4]. The aforementioned morbidities can be effectively managed with limited sequelae as long as early diagnosis and surgical treatment occur. Of note, such complications are often singular and have not been seen to present simultaneously. We present a rare case of a woman who developed a triad of post-RYGB volvulus, internal hernia and intussusception that was successfully managed via emergent exploratory laparotomy without need for bowel resection. This case is reported with consideration to the SCARE criteria [10].

## 2 Presentation of case

A 22 year old woman with a past medical history of morbid obesity treated 6 years prior with laparoscopic roux-en-y gastric bypass presented to our emergency department with a 12 h history of severe, periumbilical abdominal pain. She stated that her pain had been gradually worsening throughout the day and was now 10/10. She denied any nausea or vomiting but did admit to decrease in oral intake and decreased flatus since her last bowel movement occurring one day prior to her presentation. She denied any trauma or trigger for the pain. Her overall post-RYGB weight loss totaled 20 kg.

**Fig. 1:**
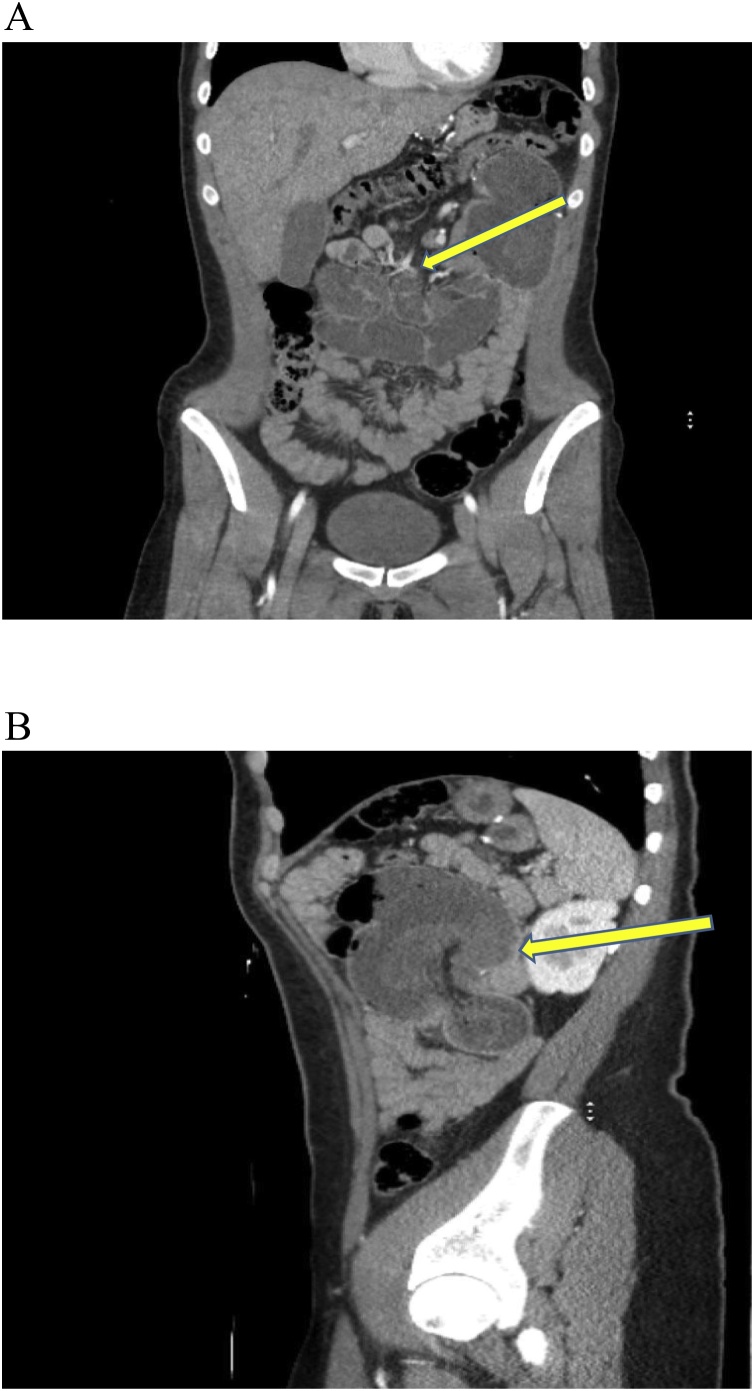
**A.** Coronal view of CT scan abdomen/pelvis showing a closed loop obstruction. in the left upper quadrant. Proximal to the obstruction, distended and dilated loops of bowel are seen with decompressed distal bowel after the obstruction site at the J-J anastomosis. The left upper quadrant mass is causing the obstruction at the J-J anastomotic site. Fig. 1**B.** Sagittal view of CT scan abdomen/pelvis showing large (grapefruit size) mass at the left upper quadrant caused by closed loop small bowel obstruction. This view shows the internal hernia where the loop of bowel herniated through the mesenteric defect at the meso-jejunal defect underneath the *J*-J anastomosis.

**Fig. 2:**
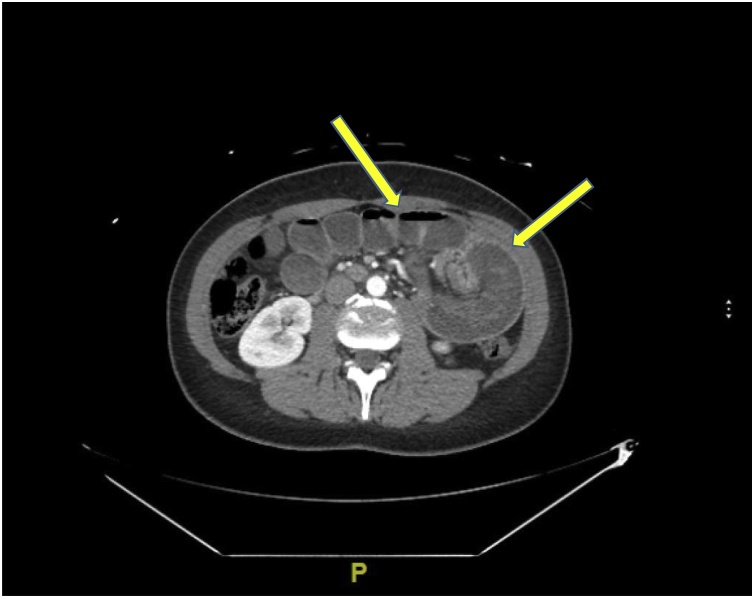
Axial CT scan of abdomen/pelvis showing small bowel obstruction with dilated loops of small bowel. Seen is a large mass in the left upper quadrant which is a closed loop bowel obstruction at the anastomotic site - J-J anastomosis. Swirling (volvulizing) of the mesentery is also seen.

Physical exam was significant for abdominal distention, decreased bowel sounds, and exquisite tenderness to palpation mainly in the left upper quadrant. Laboratory results showed mild leukocytosis (12,000) and metabolic acidosis (HCO_3_ = 21). Due to these findings, the patient was sent immediately for a CT of the abdomen and pelvis with intravenous contrast which showed findings compatible with small bowel obstruction in the left upper quadrant at the anastomotic site (Fig. 1, A–B). There were also dilated loops of small bowel present in the left upper quadrant (Fig. 2).

**Fig. 3:**
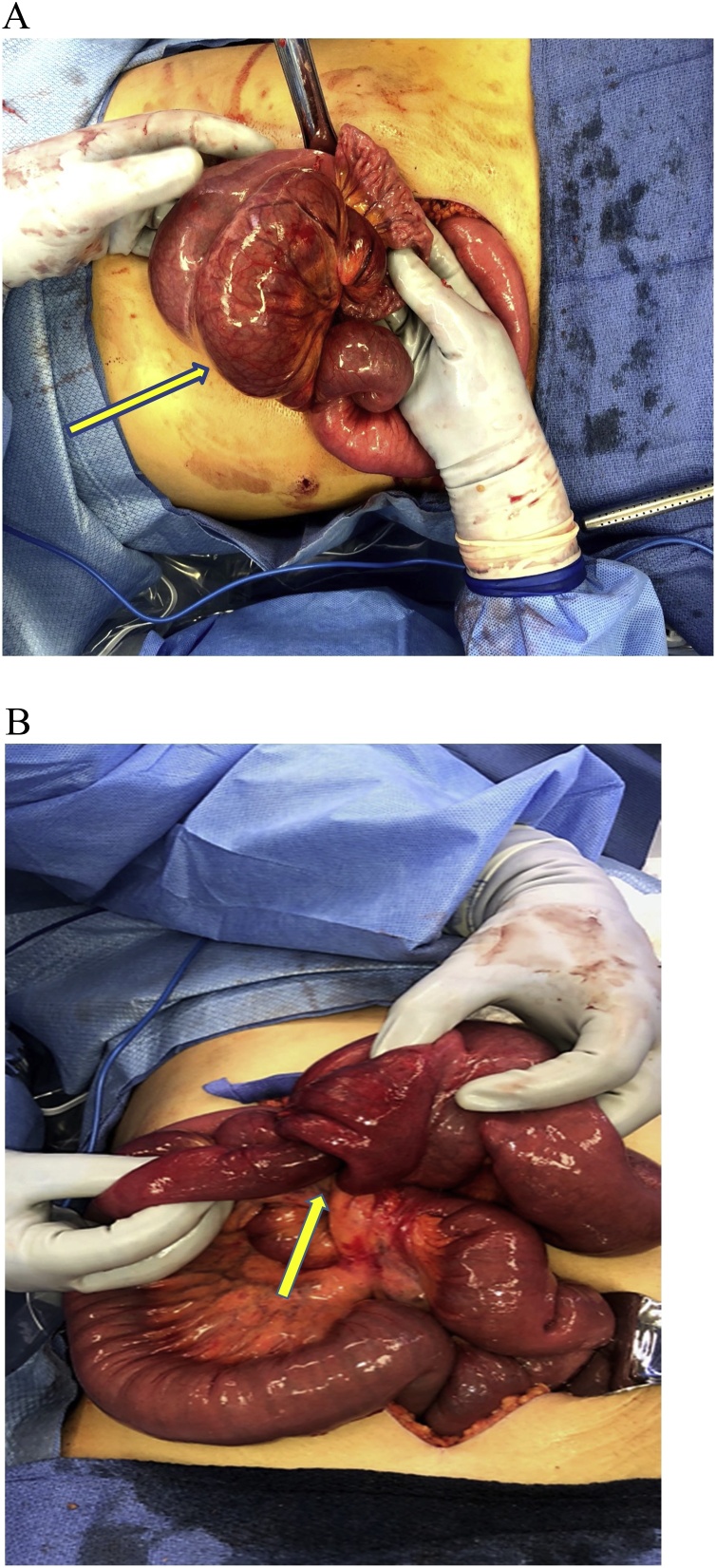
**A**. Large left upper quadrant mass. Fig. 3**B**. Intussusception.

**Fig. 4:**
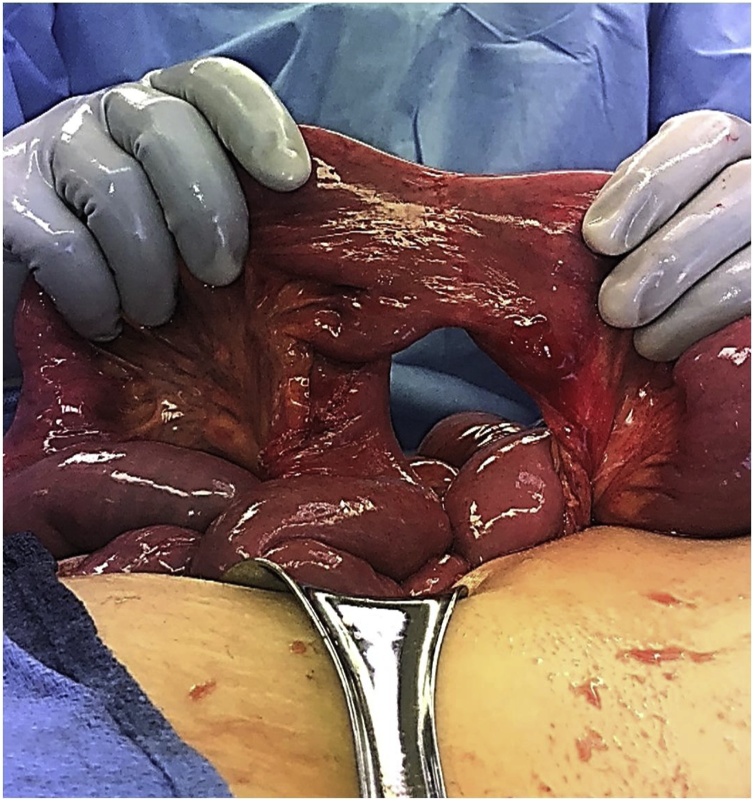
Shows the contents in the LUQ after being reduced. The reduction of the intussusception revealed a large jejunojejunal defect which is seen underneath the J-J anastomosis. The internal hernia has been mostly reduced. There is still one loop of small bowel visualized traversing the hernia defect (which was also reduced before repair of the defect).

**Fig. 5:**
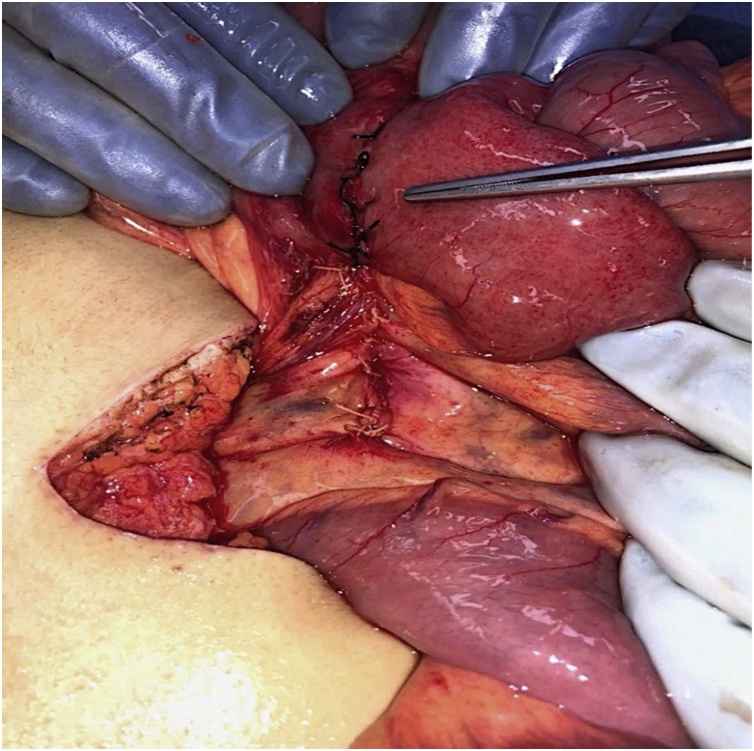
Shows the jejunojejunal defect closed nicely in order to prevent future SBO herniation at this site.

**Fig. 6:**
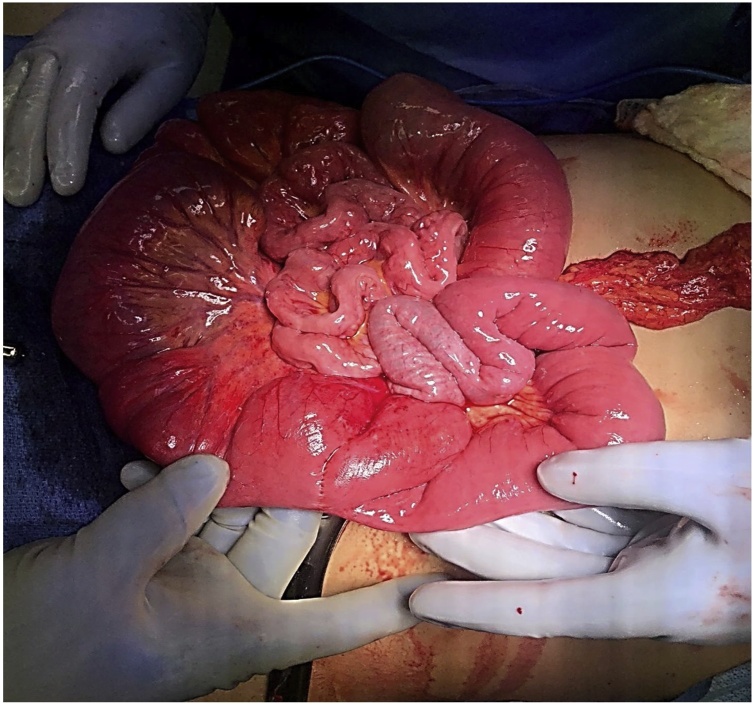
Shows the small bowel before closure of the abdomen demonstrating pink, healthy, viable bowel preventing the patients from getting small bowel resection. The J-J anastomosis is visualized and it’s pink and healthy. The patient was spared a J-J anastomotic resection and J-J reconstruction. The proximal loops of bowel were still somewhat distended from the obstruction however also pink and viable. The abdomen was then closed without bowel resection.

Diagnostic laparoscopy with expectation of surgical correction was attempted emergently due high suspicion of significant multifocal pathology based on presentation, laboratory, and imaging and concern for imminent bowel ischemia, rupture, etc. A large left upper quadrant mass of bowel was visualized but was unable to be reduced laparoscopically due to its size. After conversion to open laparotomy, a 10 × 10 cm discolored and hyperemic mass of bowel (Fig. 3A–B) was revealed at the jejunojejunal anastomoses site. Additionally, internal herniation through the jejunojejunal space, intussusception and volvulus of the small bowel at the jejunojejunal anastomoses were also noted (Fig. 4). The patient was managed with adhesion lysis, internal hernia reduction, untwisting of jejunojejunal anastomosis, reduction of jejunojejunal anastomosis intussusception, and closure of the jejunojejunal space. The mesenteric defect through which the hernia occurred was also closed as to prevent reoccurrence (Fig. 5). Once reduced, the bowel returned to a pink color with mild residual edema (Fig. 6) and peristalsis was visualized. She had an unremarkable post-operative course including a 1-day ICU stay with subsequent ward transfer. She remained on the ward for an additional 4-days prior to discharge home. Patient was noted to be doing well on outpatient follow up 2 weeks later and had no complaints or further complications.

## 3 Discussion

Intestinal complications are a rare but serious cause of morbidity in patients after RYGB surgery. Often, these complications evolve rapidly necessitating surgical resection of necrotic bowel. Such significant bowel involvement could also necessitate restructuring of the jejunojejunal anastomosis altogether, which would further worsen complications and patient outcome. Nelson et al. reviewed causes of small bowel obstruction in 784 patients who underwent RYGB and found an overall rate of 3.2 %. They also noted differences in causative factors between laparoscopic and open approaches [11]. In open RYGB, patients were more likely to have adhesive small bowel obstruction (SBO) with those who underwent laparoscopic RYGB being more likely to have obstruction at the jejunojejunostomy as seen in our patient’s case.

Imaging modalities are often insufficient in diagnosing SBO in RYGB patients due to variation in postoperative anatomy. In a study of 574 RYGB patients, CT scan was found to be falsely negative in 44–50 % of patients after diagnostic laparoscopy showed 74 % having an internal hernia and 55 % with incarcerated bowel. Three percent of those with incarcerated bowel ultimately required either bowel resection or conversion to open laparotomy [12]. In the case of our patient, an SBO was visualized on CT but could not be conclusively identified as any of the specific intestinal complications until the open laparotomy was performed. Since imaging is often insufficient in finalizing a specific diagnosis, urgent operation can decrease the risk of imminent bowel necrosis and need for bowel resection.

Intussusception occurs in only 0.6-0.7 % of patients who have undergone bariatric surgery [9]. In the general adult population, intussusception often occurs secondary to the development of a “lead point” (often a colonic mass) with subsequent telescoping of the bowel over that pathologic point. For this reason, any discovery of intussusception in this population should also involve subsequent investigation for contributing underlying pathology. However, in RYGB patients, intussusception has be shown to be rarely, if ever, associated with a lead point. The more likely mechanism behind intussusception development in this surgical population is suggested to be secondary to a motility disturbance that occurs with alteration of the jejunum and subsequent anastomotic construction [5]. Most reported cases require bowel resection due to bowel necrosis by the time the patient presents to the operating room [[11], [12], [13], [14], [15]]. One case reported a 54 year old woman with a history of RYGB presenting with abdominal pain who had confirmation of a retrograde J-J intussusception after exploratory laparoscopy. In this case, laparoscopic approach was sufficient for reduction without need for resection [16]. Our patient’s triad of intussusception, internal hernia, and volvulus was too large to accommodate adequate reduction with laparoscopic approach alone. Conversion to open laparotomy allowed for full visualization of the pathology and prevented need for bowel resection. Another case of intussusception in a 38 year old woman required bowel resection performed via a laparoscopic approach [17]. Again, the laparoscopic approach was feasible due to the presence of only an intussusception as opposed to our patient who had concomitant complications. One study reporting outcomes of repaired intussusceptions concluded that when patients are treated solely with reduction, they tend to recur. They suggested that imbrication and plication of the *J*-J anastomosis should be considered as definitive treatment for prevention of recurrent intussusception [18]. This was not performed in our patient case.

A retrospective study of volvulus occurrence showed that out of 199 cases of RYGB, only 4 cases of Roux limb volvulus were identified with all cases related to a stitch being placed between the roux limb and the gastric remnants [19]. There are very few documented cases of volvulus in this population. Another case involving cecal volvulus was found to be caused by internal herniation and required reduction and resection of the ileum and cecum [20]. The only other case with multiple small bowel complications in a patient with previous RYGB was reported in a 43 year old woman who was found to have both intussusception and volvulus. Exploratory laparotomy revealed an infarcted volvulus and intussusception of small bowel with extensive mucosal necrosis requiring resection and reconstruction [21]. This case illustrates that even without a full triad of post-RYGB SBO causes, extensive damage and necrosis to the bowel can occur rapidly and require significant, permanent intervention. Avoiding bowel resection is an obvious goal of care in patients experiencing complications from a RYGB related obstruction.

A high index of suspicion and early surgical intervention are essential in preventing bowel resections in patients with a history of RYGB presenting with signs and symptoms of SBO. In our patient’s case, she was emergently taken for CT within 60 min from presentation and ultimately to the OR within 3 h from initial presentation. Initial laparoscopy was beneficial for preliminary visualization of the intestinal defects which guided a more-focused subsequent exploratory laparotomy for adequate correction. Our timely intervention allowed for adequate reduction of her intestinal pathology without need for resection, jejunojejunal re-anastomosis, and with minimal sequela. Since volvulus and intussusception are both rare and difficult to diagnose complications of RYGB, getting the patient to the operating room as quickly as possible is of utmost importance to prevent such complications. While each individual pathology has been shown to occur in post-RYGB patients, this is, to our knowledge, the only documented case of concurrent volvulus, intussusception and internal hernia presenting as a complication of RYGB surgery. Due to rapid diagnosis and early surgical intervention, our patient did not require bowel resection and was able to be managed without additional complications.

## 4 Conclusion

To our knowledge, this is the only documented case of a triad of internal hernia, volvulus and intussusception in a patient with a history of RYGB presenting with symptoms of small bowel obstruction. Rapid surgical treatment is key to preventing bowel infarction necessitating resection which can lead to short gut syndrome, adhesions and other complications. Presentation of small bowel obstruction in a patient with a history of RYGB is variable, and while CT scans and physical examination may be helpful, the definitive diagnosis must be made intraoperatively with emergency surgery as imaging modalities are often insufficient.

## Declaration of Competing Interest

No conflicts of interest.

## Funding

This research did not receive any specific grant from funding agencies in the public, commercial or not-for-profit sectors.

## Provenance and peer review

Not commissioned, externally peer-reviewed.

## Ethical approval

This is a case report study. This report was conducted in compliance with institutional ethical standards. Informed written consent and ethical approval has been obtained and all identifying information was omitted.

## Consent

Informed written consent has been obtained and all identifying information is omitted

## Author contribution

Adel Elkbuli, Dessy Boneva, Kristen Santarone – Conception of study, acquisition of data, analysis and interpretation of data

Adel Elkbuli, Dessy Boneva, Kristen Santarone - Drafting the article

Dessy Boneva, Mark McKenney – Management of case

Adel Elkbuli, Kristen Santarone, Kyle Kinslow, Dessy Boneva, Mark McKenney – Critical revision of article and final approval of the version to be submitted

## Registration of research studies

This is a case report study.

## Guarantor

Dessy Boneva

Mark McKenney.

[22].
